# Activation-Controlled Structural Integrity in A520 MOF Membranes for Efficient CO_2_/N_2_ and CO_2_/CH_4_ Separation

**DOI:** 10.1021/acsami.6c09893

**Published:** 2026-07-08

**Authors:** Li-Tang Chi, Li-Wei Hsiao, Chia-Hui Chuang, Dun-Yen Kang

**Affiliations:** † Department of Chemical Engineering, 33561 National Taiwan University, No. 1, Sec. 4, Roosevelt Road, Taipei 106319, Taiwan; ‡ Center of Atomic Initiative for New Materials, National Taiwan University, No. 1, Sec. 4, Roosevelt Road, Taipei 106319, Taiwan; § Center of Condensed Matter Sciences, National Taiwan University, No. 1, Sec. 4, Roosevelt Road, Taipei 106319, Taiwan

**Keywords:** metal−organic framework, MOF membrane, membrane gas separation, A520, CO_2_ separation, CO_2_ capture

## Abstract

Aluminum fumarate (A520) is a promising metal–organic framework (MOF) for gas adsorption and separation. However, the fabrication of dense, pristine polycrystalline A520 membranes has not yet been reported. Herein, we present a robust seeded-growth protocol to fabricate continuous A520 polycrystalline membranes with a thickness of approximately 3 μm on α-alumina substrates. Our results demonstrate that the post-synthetic activation process plays a critical role in determining membrane integrity. In particular, a methanol exchange (ME) pretreatment prior to thermal activation effectively suppresses lattice distortion induced by capillary stress during solvent evaporation, as evidenced by X-ray diffraction and gas permeation tests. The resulting A520 (ME) membranes exhibit excellent molecular sieving performance, with ideal CO_2_/N_2_ and CO_2_/CH_4_ selectivities of 71 and 112, respectively. Mixed-gas permeation measurements reveal pronounced composition-dependent separation behavior, where the CO_2_/N_2_ separation factor reaches 153 at a 20 mol % CO_2_ feed, exceeding the 2019 Robeson upper bound, but decreases at higher CO_2_ concentrations. This behavior is associated with composition-dependent competitive adsorption and diffusion within the confined A520 channels. Grand canonical Monte Carlo (GCMC) simulations reveal that increasing N_2_ partial pressure enhances N_2_ uptake but significantly reduces its diffusional mobility, suggesting that additional N_2_ molecules occupy less favorable transport environments and experience increased diffusion resistance. In contrast, CO_2_/CH_4_ separation remains primarily governed by the intrinsic molecular sieving capability of the A520 framework, where the larger kinetic diameter of CH_4_ results in stronger diffusion limitation. These results highlight the importance of controlled activation strategies and provide insights into the interplay between competitive adsorption and confined diffusion in A520 membranes for carbon capture applications.

## Graphical abstract

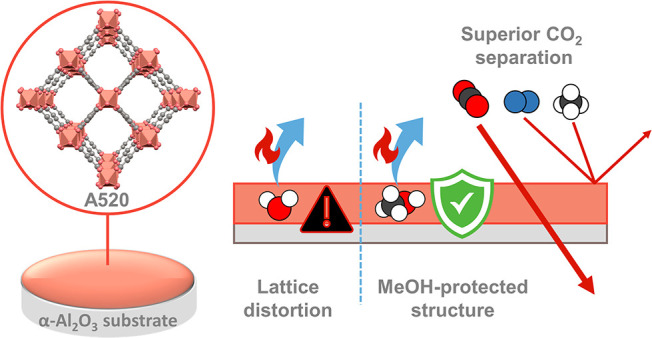

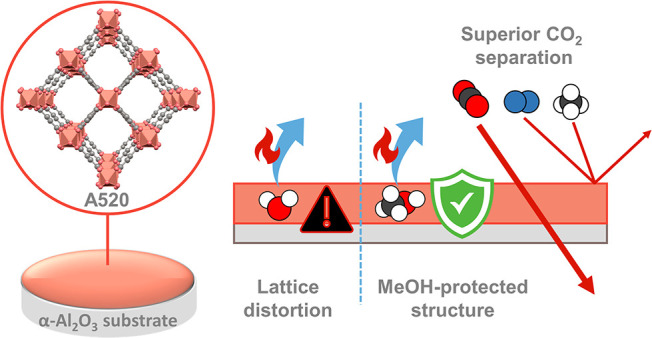

## 1 Introduction

Metal–organic frameworks (MOFs) have emerged as premier candidates for energy-efficient separations owing to their tunable porosity and chemical functionality.^1–3^ In particular, MOF-based membranes offer significant potential for CO_2_/N_2_ ^4–6^ and CO_2_/CH_4_ ^7–9^ separation, providing high-purity feedstocks for value-added chemical synthesis. Since the first report of gas-separating MOF membranes in 2009,^10,11^ research has increasingly shifted toward aluminum-based MOFs (Al-MOFs). Due to their exceptional thermal, chemical, and mechanical stabilities, Al-MOFs have demonstrated outstanding performance in diverse separation tasks, driving efforts to optimize their fabrication for large-scale industrial applications.^12^ Chi et al. developed a facile *in situ* mixed-linker strategy to fabricate MOF-303 polycrystalline membranes, achieving high ideal selectivities for CO_2_/N_2_ (76.0) and CO_2_/CH_4_ (172), comparable to those of seeded-growth counterparts but with a significantly simplified protocol.^13^ Chuang et al. successfully prepared CAU-23 membranes via a seeded-growth approach. The resulting membranes featured exceptionally fine grains and minimized intercrystalline defects, yielding CO_2_/N_2_ and CO_2_/CH_4_ selectivities of 54.3 and 69.7, respectively.^14^ Wang et al. explored the growth of MIL-160 membranes on tubular α-alumina substrates. By precisely tuning the cuboid-shaped morphology, they achieved an impressive CO_2_ permeability of 1960 Barrer with high selectivities (56.8 for CO_2_/N_2_ and 130 for CO_2_/CH_4_).^15^ Lai et al. demonstrated the versatility of Al-MOFs by fabricating ultrathin MOF-303 membranes for pervaporation. These membranes exhibited high separation factors (S.F.s) for water/ethanol (1364) and water/isopropanol (212) mixtures, with water fluxes ranging from 0.1 to 0.2 kg/m^2^-h.^16^

A520 is a premier candidate for large-scale applications due to its exceptional gas adsorption capacity^17,18^ and superior hydrothermal stability.^19,20^ Its industrial viability is particularly underscored by its cost-effective, green synthesis using fumaric acid, which is a key factor that led to its successful commercialization as Basolite A520.^19^ Consequently, extensive efforts have focused on expanding the utility of this framework across various chemical processes. BASF was the pioneer in securing patents for the A520 framework (Figure 1), providing the foundational protocols for its synthesis using aluminum ions and fumaric acid.^21^ Al-Amshany et al. further optimized the A520 synthesis by tuning reaction pH, enabling the production of high-quality micro- to nanosized powders. Notably, their protocol demonstrated the feasibility of kilogram-scale fabrication, reinforcing the material’s potential for industrial-scale production.^19^ Tan et al. utilized A520-coated aluminum foils for solar-driven atmospheric water harvesting, achieving a significant yield of 157 g/m^2^ per cycle. This underscores the material’s efficacy in high-performance, energy-efficient adsorption systems.^22^ Despite its success in powder form, fabricating continuous A520 polycrystalline membranes remains challenging due to fast nucleation kinetics and strong Al-ligand coordination. For instance, Zhao et al. attempted to grow A520 on tubular substrates but required a post-synthetic chitosan coating to seal persistent intercrystalline defects. While such repairs achieve gas-tightness, they complicate fabrication and may mask the intrinsic properties of the MOF.^23^

**Figure 1:**
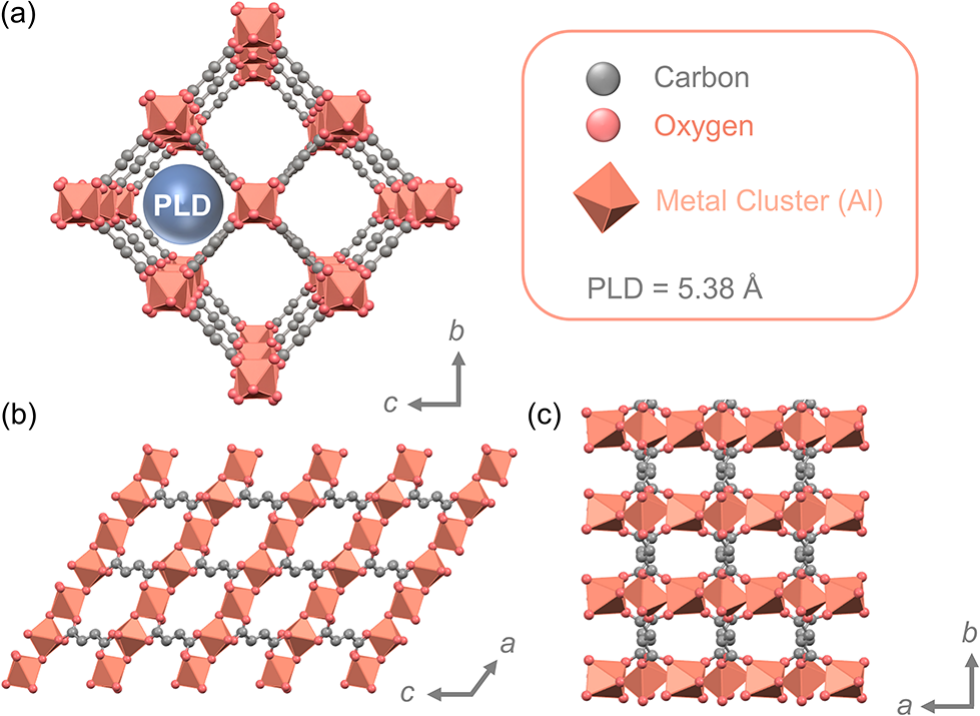
Structural representation of the A520 crystal viewed along the (a) *a*-, (b) *b*-, and (c) *c*-axes. The framework features one-dimensional channels with a pore-limiting diameter (PLD) of 5.38 Å, defined by the size of the channel aperture.

Herein, we report a robust seeded-growth protocol to fabricate dense, pristine A520 polycrystalline membranes on α-alumina substrates. By eliminating the need for secondary polymer sealing, our membranes preserve the native pore environment and rigid framework of A520, enabling a superior S.F. that directly reflects its intrinsic molecular sieving capabilities. This approach provides a pristine model for investigating the fundamental transport mechanisms of CO_2_ and other light gases through the unique pore structures of Al-fumarate. Furthermore, we identify the critical necessity of methanol exchange (ME) during the post-synthetic drying process. By replacing water with a lower-surface-tension solvent, we successfully mitigate the intense capillary forces that typically trigger drying-induced lattice distortion during solvent evaporation. This optimized drying strategy helps preserve the intrinsic structural integrity of the membrane and maintains its molecular sieving capability. Our optimized A520 membranes achieved an exceptional CO_2_/N_2_ S.F. of up to 153, marking a significant advancement in the development of high-performance, Al-MOF membranes for sustainable gas separation.

## Experimental Section

### Chemicals and Materials

Aluminum sulfate octadecahydrate (Al_2_(SO_4_)_3_·18H_2_O, ≥97%) and urea (CH_4_N_2_O, 99%) were purchased from JT Baker. Fumaric acid (C_4_H_4_O_4_, >99%), sodium hydroxide (NaOH, ≥98%), and aluminum chloride hexahydrate (AlCl_3_·6H_2_O, 99%) were purchased from Sigma-Aldrich. Methanol (CH_3_OH, 99.9%) was obtained from Merck. 3,5-pyrazoledicarboxylic acid monohydrate (C_5_H_6_N_2_O_5_, 97%) was sourced from Acros Organics. All reagents were used as received without further purification. Deionized (DI) water, produced using an ELGA VEOLIA PURELAB ultrapure water system, was used throughout all experiments.

Porous α-alumina substrates were supplied by YOMO Technology Enterprise Co. The substrates have a diameter of 40 mm and a thickness of 2 mm, and are composed of particles with an average size of approximately 300 nm and a porosity of 34% (Figure S1). Prior to membrane fabrication, the substrates were cleaned several times by sonication in DI water, followed by drying in an oven at 105 °C overnight.

### Synthesis of A520 Powder

The powder synthesis procedure was adapted from a previous study.^19^ Solution A was prepared by dissolving 3.69 g of fumaric acid and 2.71 g of sodium hydroxide in 54.6 g of DI water. The mixture was placed in a sealed Teflon container, continuously stirred, and heated on a hot plate at 90 °C.

Solution B was prepared by dissolving 10.6 g of aluminum sulfate octadecahydrate in 45.4 g of DI water, also in a sealed Teflon container under the same stirring and heating conditions. After both solutions were stirred and heated for 1 h to ensure complete dissolution, Solution A was rapidly poured into Solution B under continuous stirring. The resulting mixture was maintained at 90 °C with stirring for 2 h, and then allowed to cool to room temperature.

The resulting white powder was collected by centrifugation at 8500 rpm for 5 min. The product was washed five times with DI water, using the same centrifugation conditions each time to remove the supernatant. Finally, the powder was dried in an oven at 90 °C overnight and gently ground into a fine powder using a mortar.

### Synthesis of MOF-303 Powder

MOF-303 powders were synthesized as a comparative Al-based MOF following a previously reported protocol.^16^ Briefly, a urea stock solution was prepared by dissolving 3.9 g of urea in 30 g of DI water. Separately, 0.722 g of aluminum chloride hexahydrate and 0.521 g of 3,5-pyrazoledicarboxylic acid monohydrate were dissolved in 100 mL of DI water. Subsequently, 2.65 mL of the urea solution was added to the aluminum precursor solution. The mixture was then refluxed at 110 °C for 16 h under vigorous stirring. The resulting MOF-303 powders were collected via vacuum filtration, washed thoroughly with DI water, and dried at 100 °C overnight.

### Synthesis of A520 Polycrystalline Membrane

A seeding suspension was prepared by dispersing 0.015 g of A520 powder in 5 mL of DI water, followed by sonication for 5 min to obtain a uniform suspension. An α-alumina substrate was then placed on a Laurell spin coater (Model WS-650MZ-23NPPB), and approximately 1.5 mL of the seeding suspension was dropped onto the substrate. After allowing 10 s for the particles to settle, the substrate was spin-coated at 3000 rpm for 30 s. This spin-coating process was repeated three times to ensure uniform seed coverage. The seeded substrate was subsequently dried in an oven at 105 °C for 30 min.

The secondary growth solution was prepared by dissolving 1.49 g of aluminum sulfate octadecahydrate and 0.26 g of fumaric acid in 50 mL of DI water. The solution was sonicated in a 40 °C ultrasonic bath for 10 min to ensure complete dissolution. The seeded substrate was fixed upside down using a Teflon clamp and then immersed in the solution in a 225 mL Teflon-lined autoclave. The autoclave was placed in a preheated oven at 100 °C for 10 h to allow membrane growth.

Following the hydrothermal synthesis, the autoclave was rapidly cooled down to room temperature in a continuous-flow water bath. The as-synthesized membranes were removed and thoroughly rinsed several times with DI water to remove any residual reactants from the surface. To investigate the impact of the activation process on membrane integrity, two distinct drying protocols were employed:

A520 (as made): The water-rinsed membranes were directly placed in a preheated oven and dried at 105 °C for 1 h.

A520 (ME): The membranes were immersed in methanol for 30 min to facilitate the exchange of pore-confined water with methanol. This exchange procedure was repeated three times, with the methanol refreshed every 30 min. Subsequently, the membranes were dried in a preheated oven at 105 °C for 1 h. These samples were designated as A520 (ME).

### Materials Characterization

Scanning electron microscopy (SEM) was performed using a Hitachi S-4800 field-emission scanning electron microscope (FE-SEM) to investigate the morphology of both powder and membrane samples. Before imaging, all samples were coated with a thin layer of gold via sputtering at 35 mA for 30 s. The observations were carried out at an accelerating voltage of 10 kV.

N_2_ adsorption at 77 K, CO_2_ adsorption at 273 K, and single-component adsorption isotherms of CO_2_, N_2_, and CH_4_ at 308 K were measured using a TriStar II Plus analyzer. Prior to the first measurement, the powder sample was degassed under vacuum at 150 °C for 800 min to remove adsorbed species. Between consecutive adsorption measurements, the sample was reactivated by degassing under vacuum at 150 °C for 120 min.

Thermogravimetric analysis (TGA) was conducted using a TA Instruments SDT 650 system. The sample was first equilibrated at 35 °C, then heated to 800 °C at a constant rate of 10 °C min^–1^. The analysis was performed under an air atmosphere with a flow rate of 100 mL/min.

X-ray diffraction (XRD) analysis was carried out using a Rigaku SmartLab SE diffractometer with Cu Kα radiation (λ = 1.5418 Å). Diffraction patterns for both powder and membrane samples were collected over a 2θ range of 5–50°, using a step size of 0.01° and a scanning speed of 4° min^–1^.

### Membrane Gas Permeation

Membrane gas permeation experiments were carried out using the constant-volume method with a custom-designed setup (Figure S2). For single-gas measurements, the membrane was installed in a stainless-steel cell and sealed using aluminum foil tape together with an O-ring. To minimize potential leakage, epoxy resin (3 M DP-100) was further applied at the interface between the membrane edge and the aluminum tape. The membrane module was placed inside a convection oven to maintain a constant operating temperature during the permeation measurements. Prior to each experiment, the membrane was activated under vacuum (<50 mTorr) at 150 °C for 8 h using a heat tape wrapped around the membrane module. The heat tape enabled localized heating of the membrane while minimizing heat exposure to the tubing, valves, and other components of the permeation system, thereby preventing thermal damage. After activation, the system was isolated from the vacuum source to begin the permeation experiment.

In a typical single-gas permeation test, the target gas (H_2_, CO_2_, N_2_, or CH_4_) was fed to the upstream side at an absolute pressure of 3 bar, while the temperature was maintained at 308 K. As gas permeated through the MOF membrane, the downstream pressure gradually increased and was continuously recorded using a pressure transducer (MKS AA09A12TCE0). The gas permeability was determined using the following equation

1 $$P_{i}=\frac{V}{\mathrm{RT}}\frac{l}{A\Delta p_{i}}\left(\frac{dp_{i}}{dt}\right)$$

where *P_i_* is the permeability of component *i*, *l* is the membrane thickness (obtained from SEM images), *A* is the effective membrane area, and *V* is the permeate-side volume. Δ*p_i_* represents the transmembrane pressure difference of component *i* (approximated as 3 bar due to the negligible downstream pressure), *R* is the gas constant, and *T* is the absolute temperature. The term (d*p_i_*/d*t*) corresponds to the rate of pressure rise on the permeate side.

For mixed-gas experiments, binary mixtures of CO_2_/N_2_ or CO_2_/CH_4_ with varying compositions (*X*_CO_2__ = 0.2, 0.5, and 0.8) were used as the feed at a total pressure of 3 bar. These tests followed the same procedure as the single-gas measurements, including membrane activation and permeation analysis. The composition of the permeate stream was analyzed using a gas chromatograph (Shimadzu GC-2014). Argon was used as a carrier gas to transport permeated species to the gas chromatograph. The system was equipped with a thermal conductivity detector and a Shincarbon-ST column for accurate separation and quantification. The S.F. for a binary mixture was calculated according to

2 $$S.F.=\frac{y_{i}/y_{j}}{x_{i}/x_{j}}$$

where *x_i_* and *y_i_* denote the molar fractions of component *i* in the feed and permeate streams, respectively, and *x_j_* and *y_j_* correspond to those of component *j*.

### Molecular Simulation

Binary gas adsorption in A520 was simulated using the open-source RASPA^24^ software package based on the grand canonical Monte Carlo (GCMC) method. The crystal structure of A520 was obtained from the Cambridge Crystallographic Data Centre (CCDC, deposition number 1051975), with all guest water molecules removed prior to simulation. To maintain computational efficiency, the A520 framework and all gas molecules were treated as rigid bodies. Intermolecular interactions were modeled using a combination of the 12–6 Lennard-Jones (L-J) potential and Coulombic electrostatic forces, applying a cutoff distance of 12 Å. The L-J parameters for the framework atoms (C, H, O, and Al) were adopted from the DREIDING force field,^25^ while CO_2_,^26^ N_2_,^26^ and CH_4_ ^27^ molecules were described using the TraPPE force field. Parameters for interactions between dissimilar atoms were determined via the Lorentz–Berthelot mixing rules. To ensure accurate electrostatic descriptions, atomic partial charges for the A520 framework were assigned using the multilayer connectivity-based atom contribution (m-CBAC) approach.^28^ Electrostatic interactions were calculated using the Ewald summation method. All simulations were conducted at 308 K (35 °C) and a total pressure of 3 bar to match experimental conditions. For each binary mixture (CO_2_/N_2_ and CO_2_/CH_4_), each adsorption data point was obtained through 200,000 GCMC cycles.

## Results and Discussion

A520 powder was synthesized for use as a seed layer in membrane fabrication and for subsequent characterization. The obtained A520 powder exhibits a uniform particle size distribution centered at approximately 50 nm, as shown in Figure S3a. As illustrated in Figure S3b, the experimental pattern is in good agreement with the simulated structure derived from the crystallographic information file (CIF) from CCDC #1051975 with removal of water, confirming the successful synthesis of A520 with the expected crystal structure. TGA was then performed on A520 and MOF-303 powders for comparison, as shown in Figure S3c. A rapid weight loss is observed between 50 and 75 °C for A520, which can be attributed to the removal of physically adsorbed water. In comparison, MOF-303 exhibits a similar weight-loss step between 60 and 90 °C, occurring at slightly higher temperatures and with a greater mass loss than that of A520. This behavior may be attributed to the pyrazole-based linker in MOF-303, which gives the framework a stronger affinity for water due to its ability to form hydrogen bonds.^16^ Consequently, MOF-303 adsorbs more water and requires a higher temperature for water desorption. A second significant weight-loss event is observed at approximately 500 °C for both MOFs, corresponding to the decomposition of the organic linkers. These results demonstrate that A520 possesses excellent thermal stability, as indicated by its relatively high decomposition temperature.

Pore characteristics were investigated with N_2_ adsorption at 77 K and CO_2_ adsorption at 273 K. The N_2_ adsorption isotherm at 77 K (Figure S4a) shows that A520 reaches a maximum adsorption capacity of approximately 10.5 mol/kg. This result is in excellent agreement with previously reported values for high-quality A520,^17^ confirming the successful synthesis of a high-purity crystalline framework without significant pore blockage. Specifically, the observed capacity exceeds that of MIL-160 but remains slightly lower than that of MOF-303, consistent with the documented hierarchy of surface areas for these Al-MOF.^13^ The CO_2_ adsorption isotherm (Figure S4b) reveals an uptake of about 4.80 mol/kg at 1 bar, and this enhanced CO_2_ adsorption capacity may contribute to the superior membrane permeance discussed later. The pore size distribution was further analyzed using the CO_2_ adsorption isotherm at 273 K via a density functional theory (DFT) model (Figure S4c). The pore sizes are primarily distributed in the range of 5–7 Å, with a peak at approximately 6 Å. This result is in good agreement with the largest cavity diameter of 5.83 Å calculated from the CIF structure file using Zeo++.^29–31^ These findings suggest that the synthesized A520 possesses well-activated and accessible pores, with minimal residual water or unreacted species.

The gas adsorption isotherms of CO_2_, N_2_, and CH_4_ in A520 at 308 K are presented in Figure 2, with MOF-303 powder included as a benchmark. Both A520 and MOF-303 exhibit a significantly higher uptake of CO_2_ compared to N_2_ and CH_4_, although MOF-303 demonstrates a substantially superior adsorption capacity. Specifically, at 1 bar, A520 achieves a CO_2_ uptake of 1.86 mol/kg, whereas MOF-303 reaches 5.27 mol/kg. This disparity is attributed to the presence of pyrazole-based linkers in MOF-303, which can form strong dipole–quadrupole interactions with CO_2_ molecules, whereas the fumarate linkers in A520 exhibit a lower affinity toward CO_2_. The enhanced surface chemistry of MOF-303 also translates into higher ideal adsorption selectivity. The CO_2_/N_2_ selectivity for MOF-303 is 21.3, nearly double that of A520 (11.9). Similarly, the CO_2_/CH_4_ selectivity is 5.70 for MOF-303 compared to 3.10 for A520. These results suggest that the inherently lower adsorption selectivity of A520 may limit its separation efficiency in mixed-gas permeation tests, as the competitive adsorption effects are less pronounced than in MOF-303.

**Figure 2:**
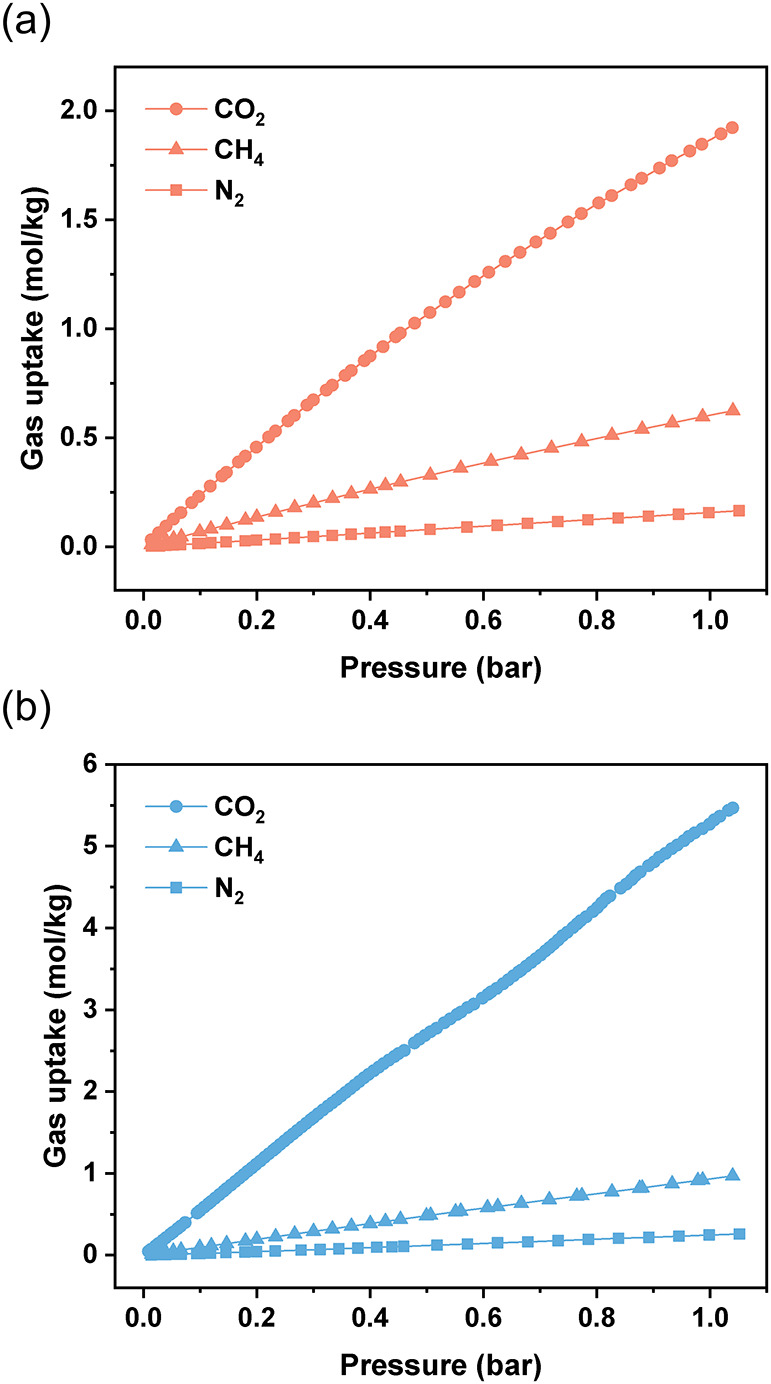
Single-component adsorption isotherms of CO_2_, N_2_, and CH_4_ measured at 308 K for (a) A520 and (b) MOF-303 powders.

A520 membranes were fabricated via the seeded-growth method. A critical finding in this work is that the ME process is indispensable for maintaining membrane structural integrity.^32,33^ To investigate the impact of activation conditions on membrane integrity, two distinct drying protocols were employed following the synthesis. The first set, referred to as A520 (as made), was rinsed several times with DI water and directly dried in a preheated oven at 105 °C for 1 h without any solvent exchange. The second set, denoted as A520 (ME), underwent an ME procedure prior to activation. These membranes were immersed in methanol for 30 min (repeated three times with fresh solvent) before being dried at 105 °C for 1 h. All other synthesis and activation parameters remained identical to ensure a controlled comparison of the drying-induced effects on the framework structure. Figure 3a shows the surface and cross-sectional SEM images of the A520 (ME) membrane. The top-view SEM image reveals a densely packed layer of cubic crystals with good intergrowth between adjacent grains, and no obvious defects or pinholes are observed. Cross-sectional SEM analysis confirms the formation of a dense and thin membrane with a thickness of approximately 3 μm. For comparison, Figure 3b presents the surface and cross-sectional SEM images of the A520 (as made) membrane. Both the A520 (ME) and A520 (as made) membranes exhibit similar surface morphologies, characterized by well-intergrown cubic crystals, as well as comparable membrane thicknesses. These observations suggest that the two membranes are morphologically similar and that any structural differences between them cannot be distinguished solely from SEM analysis.

**Figure 3:**
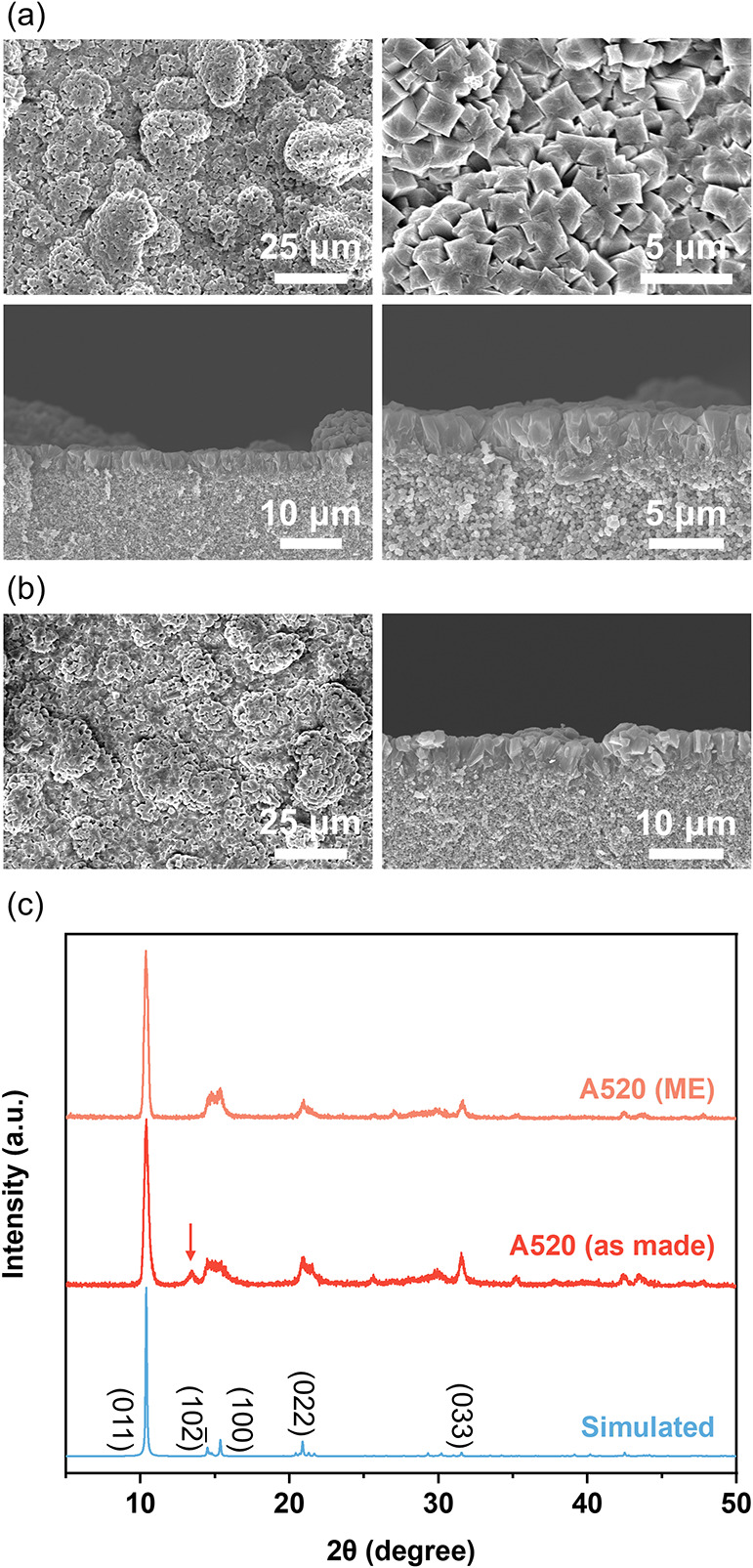
Top-view and cross-sectional SEM images of the (a) A520 (ME) and (b) A520 (as made) membranes. (c) Comparison of the experimental XRD patterns of the two A520 membranes with the simulated powder diffraction pattern. The red arrow indicates an additional diffraction peak observed in the A520 (as made) membrane, which may originate from localized structural distortion induced by capillary stress during water evaporation.

However, distinct structural differences are observed in the XRD patterns (Figure 3c). The A520 (as made) membrane exhibits a prominent additional diffraction peak at 2θ ≈ 13.5°, a feature previously attributed to lattice distortion or structural deformation.^34,35^ According to Bragg’s law, this peak corresponds to a *d*-spacing of approximately 6.55 Å. This lattice distortion is expected to significantly impact gas separation performance by disrupting the intrinsic molecular sieving channels of the A520 framework, as discussed in the following sections. In contrast, the A520 (ME) membrane aligns perfectly with the simulated pattern, confirming the formation of a high-purity and structurally intact A520 phase.

To further investigate the origin of this additional diffraction peak, a controlled dehydration experiment was performed (Figure 4). A freshly synthesized A520 membrane was first characterized by XRD immediately after removal from the autoclave and rinsing with DI water. The as-synthesized membrane exhibited a well-defined diffraction pattern, and no additional reflection was observed in the region around 2θ ≈ 13.5°, indicating that this feature was not an intrinsic characteristic of the pristine hydrated A520 framework, nor was it associated with residual reactants or synthesis-derived impurities. The same membrane was subsequently subjected to thermal activation at 105 °C for 1 h, followed by XRD characterization under identical conditions. After thermal dehydration, a distinct new diffraction peak emerged at 2θ ≈ 13.5°. The fact that this reflection appeared only after water removal demonstrates that the peak was generated during the dehydration process rather than originating from the initial synthesized structure. In addition, the absence of this reflection in the as-synthesized hydrated membrane and its appearance after activation suggest that the peak does not correspond to another hydrated phase or a partially collapsed framework structure. Instead, the emergence of this additional diffraction feature is more likely associated with a localized structural rearrangement or lattice distortion induced by capillary stress during thermal dehydration. This structural evolution reflects a deformation of the framework at the local scale rather than the formation of a new crystalline phase.

**Figure 4:**
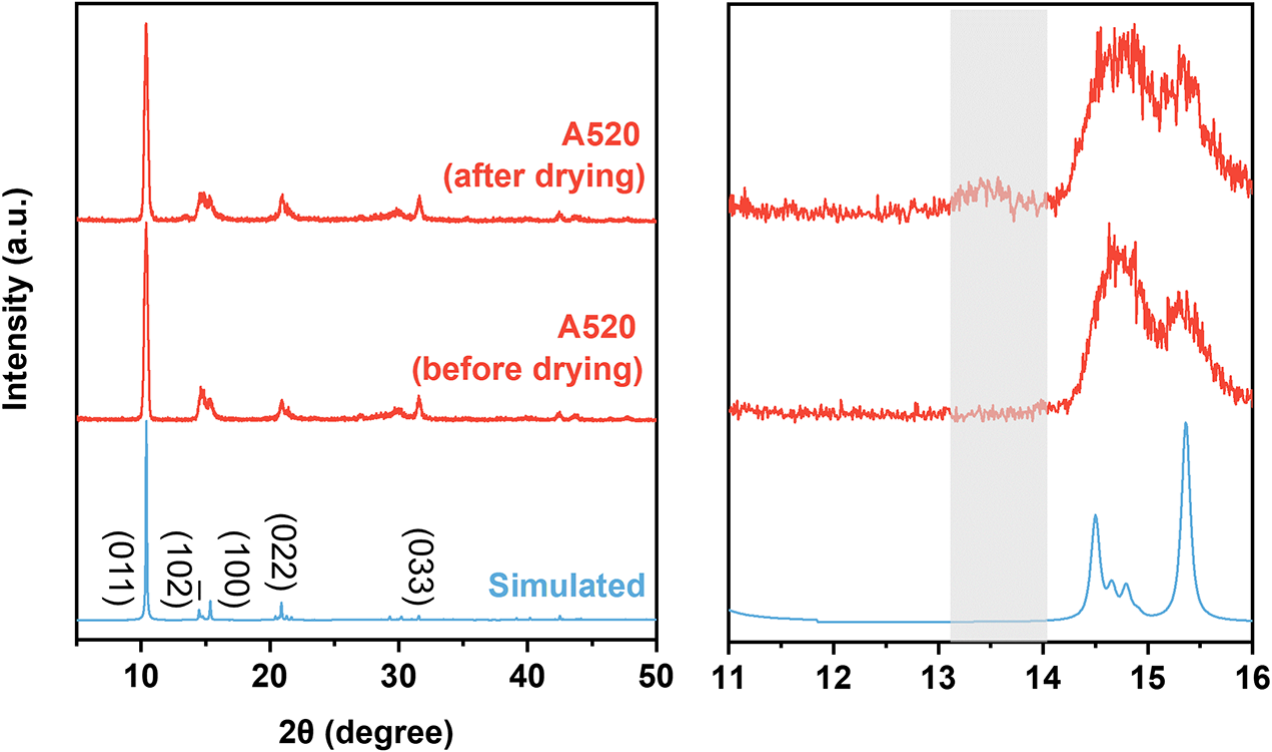
XRD patterns of the A520 membrane before and after the drying process. The as-synthesized membrane, characterized immediately after hydrothermal synthesis, does not exhibit the additional diffraction peak at 2θ ≈ 13.5°. In contrast, this peak emerges after direct thermal activation. These results suggest that the additional reflection is induced during the dehydration process and is associated with localized structural distortion rather than impurity phases, highlighting the importance of ME treatment for preserving the framework structure.

The single-gas permeation performance is presented in Figure 5. The gas permeation properties of a previously reported MOF-303 membrane^13^ are also included for comparison. The H_2_ and CO_2_ permeabilities of the three membranes are of similar magnitude. For A520 (ME), the H_2_ and CO_2_ permeabilities are 249 and 67.2 Barrer, respectively, whereas those of A520 (as made) are 120 and 23.8 Barrer, and those of MOF-303 are 145 and 30.3 Barrer. The relatively moderate H_2_ and CO_2_ permeabilities observed for all three membranes suggest a continuous and well-intergrown layer, effectively minimizing nonselective transport pathways such as large pinholes or cracks. However, a distinct trend is observed for N_2_ and CH_4_ permeation. The A520 (as made) membrane exhibits significantly higher permeabilities for N_2_ and CH_4_, with values of 29.6 and 38.8 Barrer, respectively, which even exceed its CO_2_ permeability. This behavior indicates a loss of molecular sieving ability and suggests that gas transport in A520 (as made) follows a Knudsen diffusion mechanism. In contrast, A520 (ME) demonstrates markedly improved separation performance, with very low N_2_ and CH_4_ permeabilities of 0.947 and 0.602 Barrer, respectively. These values are comparable to those of MOF-303 (0.489 and 0.228 Barrer, respectively), indicating effective molecular sieving. As a result, A520 (ME) achieves excellent ideal selectivities, with H_2_/N_2_ = 263, H_2_/CH_4_ = 414, CO_2_/CH_4_ = 112, and CO_2_/N_2_ = 71.0. These values are also comparable to those of MOF-303 (297, 636, 133, and 62.0, respectively), demonstrating the high separation performance of the A520 (ME) membrane.

**Figure 5:**
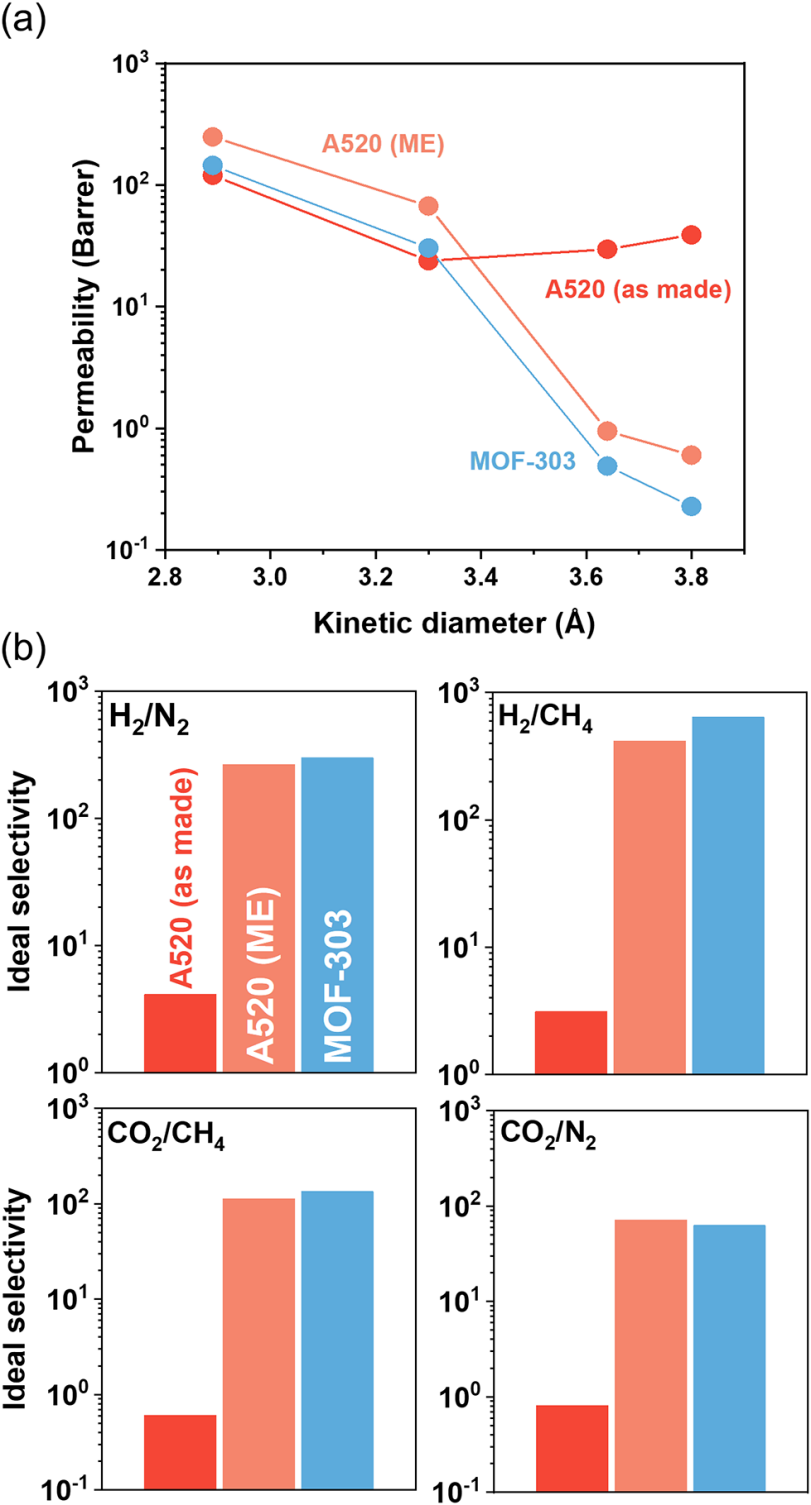
Single-gas permeation performance of A520 and MOF-303 membranes measured at 308 K and 3 bar. (a) Gas permeabilities of H_2_, CO_2_, N_2_, and CH_4_ for the A520 (ME) and A520 (as made) membranes, with MOF-303 included for comparison. (b) Ideal selectivities for H_2_/N_2_, H_2_/CH_4_, CO_2_/N_2_, and CO_2_/CH_4_.

The performance disparity between A520 (ME) and A520 (as made) can be attributed to lattice distortions triggered by intense capillary stresses during the direct thermal activation of the as-made samples. In the case of A520 (as made), the absence of solvent exchange leaves the pores filled with water. During drying, the high surface tension of water (60.22 mN/m at 100 °C)^36^ generates significant capillary stress as it evaporates. This stress may induce localized lattice distortion and structural rearrangement, resulting in modified gas transport pathways and reduced molecular sieving capability of the membrane. These lattice distortions may introduce additional nonselective transport pathways, facilitating the diffusion of larger molecules such as N_2_ and CH_4_ and thereby reducing the molecular sieving capability of the membrane. Conversely, for the A520 (ME) membrane, the intrapore water is completely replaced by methanol. The significantly lower surface tension and vaporizing force of methanol (18.63 mN/m at 60.6 °C)^37^ prevent the formation of drying-induced lattice distortion, thereby preserving the structural integrity and the intrinsic molecular sieving effect of the A520 framework. Interestingly, while the separation factor drops significantly for A520 (as made), the fluxes of H_2_ and CO_2_ remain comparable to those of A520 (ME). This phenomenon occurs because H_2_ and CO_2_, with their smaller kinetic diameters, already permeate rapidly through the original A520 pores. The emergence of drying-induced lattice distortion provides additional voids for transport, but since these small molecules are not significantly restricted by the framework to begin with, their total flux does not show a magnitude-order increase. However, for larger molecules (N_2_ or CH_4_), this lattice distortion eliminates the steric hindrance previously imposed by the A520 apertures. Consequently, their permeance increases disproportionately, shifting the transport mechanism toward Knudsen diffusion and leading to the observed collapse in selectivity.

To evaluate the separation performance under practical operating conditions, mixed-gas permeation tests for CO_2_/N_2_ and CO_2_/CH_4_ mixtures were conducted at 308 K (Figure 6a,b). For the CO_2_/N_2_ system, as the CO_2_ concentration in the feed increased from 20% to 80%, the CO_2_ permeability decreased from 111.74 to 44.19 Barrer, while the N_2_ permeability rose from 0.73 to 2.37 Barrer. Consequently, the CO_2_/N_2_ S.F. declined sharply from 153.3 to 18.6. To identify the parameters governing this behavior, GCMC simulations were employed to calculate competitive solubilities (*S*) at 308 K and a total pressure of 3 bar (Figure 6c and d). The corresponding diffusivities (*D*) were subsequently estimated by dividing the experimentally measured permeabilities (*P*) by the simulated solubilities according to the solution–diffusion model (*P* = *S* × *D*).^38^ The calculated CO_2_ and N_2_ solubilities and diffusivities in A520 are presented in Figure 6c–f. It should be noted that a framework density of 1060 kg/m^3^, calculated from the CIF using Mercury software, was used to convert the simulated adsorption loadings into solubility values.

**Figure 6:**
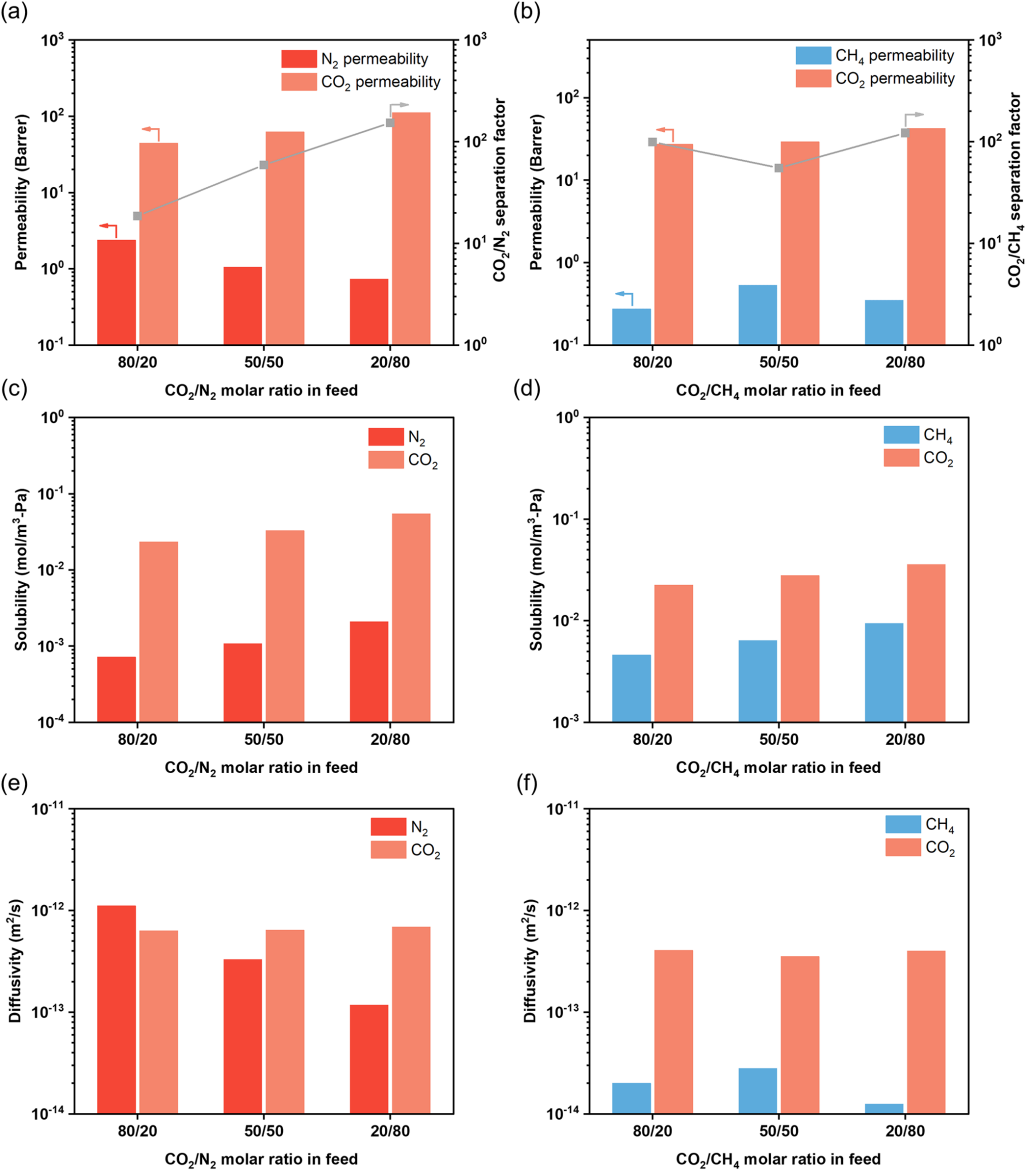
Mixed-gas permeation performance of A520 and MOF-303 membranes. Mixed-gas permeabilities and separation factors as a function of feed composition for (a) CO_2_/N_2_ and (b) CO_2_/CH_4_ mixtures measured at 308 K and 3 bar. GCMC-simulated competitive gas solubilities in A520 for (c) CO_2_/N_2_ and (d) CO_2_/CH_4_ mixtures at 308 K and a total pressure of 3 bar. Gas diffusivities for (e) CO_2_/N_2_ and (f) CO_2_/CH_4_ calculated from the experimental permeability and simulated solubility data based on the solution–diffusion model.

The analysis reveals that the decline in CO_2_ permeability with increasing CO_2_ partial pressure is primarily a solubility-driven phenomenon. The CO_2_ solubility decreases as the CO_2_ feed ratio rises, which aligns with the thermodynamic characteristics of Langmuir-type adsorption where solubility is highest at the low-pressure (Henry’s law) region and gradually declines as adsorption sites approach saturation. Notably, the CO_2_ diffusivity remains relatively stable across the entire concentration range, indicating that the transport of the strongly adsorbing CO_2_ is not significantly hindered by the presence of other species. In contrast, the transport of N_2_ is highly sensitive to the gas composition. Specifically, as the N_2_ partial pressure increases from 0.6 to 2.4 bar, the mixed-gas permeability decreases from 2.37 to 0.73 Barrer, while its solubility increases from 7.13 × 10^–4^ to 2.08 × 10^–3^ mol/m^3^-Pa. Consequently, the calculated diffusivity significantly decreases from 1.11 × 10^–12^ to 1.17 × 10^–13^ m^2^/s. These results demonstrate that the reduction in permeability of N_2_ is governed primarily by suppressed diffusional mobility.

For a more direct evaluation of the changes in gas loading and adsorption-site occupancy, the GCMC-calculated mixed-gas solubility coefficients were converted into adsorption uptake values. The uptake of each gas component as well as the total adsorption uptake are presented in Figure S5. As the N_2_ partial pressure increased from 0.6 to 2.4 bar, the CO_2_ uptake decreased from 5.29 to 3.09 mol/kg, whereas the N_2_ uptake increased from 4.04 × 10^–2^ to 4.71 × 10^–1^ mol/kg. Because the reduction in CO_2_ uptake was substantially larger than the increase in N_2_ uptake, the total adsorption uptake decreased from 5.33 to 3.56 mol/kg. These results confirm the stronger adsorption affinity of CO_2_ toward the A520 framework under mixed-gas conditions and provide a more intuitive description of the adsorption behavior than solubility coefficients alone.

Therefore, the simultaneous increase in solubility and decrease in permeability can be consistently interpreted as a pronounced reduction in diffusivity. Although the total adsorption uptake decreases with increasing N_2_ partial pressure (Figure S5a), the diffusivity of N_2_ decreases by nearly 1 order of magnitude, whereas that of CO_2_ remains largely unchanged. This behavior suggests that N_2_ transport is strongly influenced by the mixed-gas adsorption environment within the A520 framework. As the N_2_ loading increases, additional N_2_ molecules may occupy less favorable adsorption configurations or more confined regions near the diffusion bottlenecks, resulting in increased resistance for molecular hopping between adjacent adsorption sites. Meanwhile, the preferential adsorption of CO_2_ maintains a distinct local adsorption environment that affects the distribution and mobility of weakly adsorbed N_2_ molecules. Consequently, the averaged N_2_ diffusivity decreases substantially, leading to reduced N_2_ permeability.

A similar but more pronounced trend was observed in the CO_2_/CH_4_ mixed-gas system. The separation factor exhibited a nonmonotonic variation, increasing to 99.4 at an 80% CO_2_ feed. To better understand this behavior, the mixed-gas adsorption results were further analyzed in terms of gas uptake (Figure S5b). As the CH_4_ partial pressure increased, the CH_4_ uptake increased, whereas the CO_2_ uptake decreased more substantially, resulting in a continuous decrease in total adsorption uptake. This confirms the stronger adsorption affinity of CO_2_ toward the A520 framework under competitive conditions. Despite the increased CH_4_ uptake, its diffusivity exhibited a nonmonotonic variation, indicating that CH_4_ transport is governed primarily by diffusion rather than adsorption capacity. Due to its larger kinetic diameter (3.8 Å) compared with N_2_ (3.64 Å), CH_4_ is more sensitive to the local adsorption environment and confinement effects within the rigid microporous channels. Consequently, the preferential retention of CO_2_ together with the size-selective diffusion restriction of CH_4_ leads to enhanced CO_2_/CH_4_ separation performance.

To evaluate the reliability of the simulation, adsorption isotherms of CO_2_, N_2_, and CH_4_ in A520 were calculated at 35 °C up to 3 bar and compared with the corresponding experimental data (Figure S6). The results show that both GCMC simulations and experimental measurements exhibit the same adsorption affinity trend, following the order of CO_2_ > CH_4_ > N_2_, demonstrating that the simulation successfully captures the relative adsorption selectivity of the A520 framework. Although the simulated adsorption capacities are higher than the experimental values, likely due to the idealized framework model and force-field limitations, the difference mainly affects the absolute values of the calculated transport parameters. The higher simulated uptake results in larger calculated solubility coefficients, which can influence the diffusivity values obtained from the solution–diffusion model. Nevertheless, both simulation and experimental results consistently reveal the same adsorption hierarchy and composition-dependent transport behavior in the A520 framework.

The separation performance of the A520 (ME) membrane is benchmarked against current state-of-the-art materials using the Robeson plots (Figure 7a,b), which represent the empirical trade-off limits primarily derived from polymeric materials. As shown in the plots, a stark contrast is observed between A520 (as made) and A520 (ME). The former exhibits performance far inferior to previously reported materials due to its poor ideal selectivity. Conversely, the A520 (ME) membrane demonstrates exceptional performance, slightly surpassing MOF-303 and closely approaching the 2008 and 2019 upper bounds for CO_2_/N_2_ and CO_2_/CH_4_, respectively.^39,40^ To provide a comprehensive evaluation, the performance of several representative MOF membranes reported in the literature is also included for comparison,^9,13,41–56^ with detailed data and corresponding references summarized in Tables S1 and S2.

**Figure 7:**
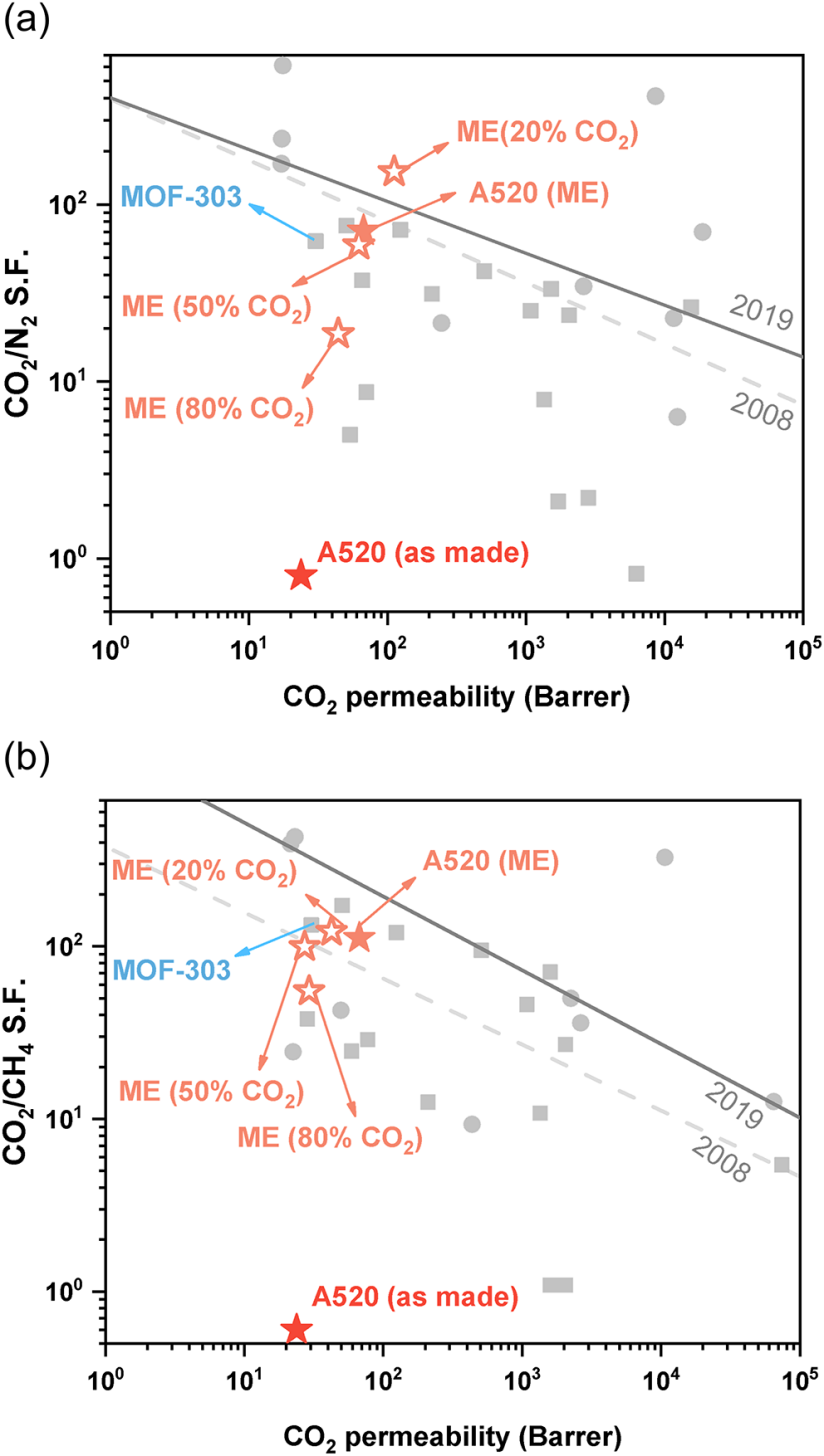
Robeson plots for (a) CO_2_/N_2_ and (b) CO_2_/CH_4_ separations. Literature-reported MOF membranes are represented by circles and cubes for mixed-gas and single-gas permeation data, respectively. Filled and open star symbols indicate single-gas and mixed-gas measurements for A520 and MOF-303 membranes, respectively. For the single-gas permeation data, the ideal selectivity is used as the separation factor (S.F.) in the plots. The corresponding literature data are summarized in Tables S1 and S2.

Notably, under mixed-gas conditions with a low CO_2_ feed molar ratio (20%), the CO_2_/N_2_ S.F. of the A520 (ME) membrane surpasses the 2019 upper bound. This significant enhancement compared with the single-gas results arises from the distinct adsorption–diffusion interplay under competitive mixed-gas conditions. Although N_2_ uptake increases with increasing N_2_ partial pressure, its diffusivity decreases substantially, leading to a pronounced suppression of N_2_ permeation. In contrast, the diffusivity of CO_2_ remains relatively unaffected due to its strong preferential adsorption within the A520 framework. As a result, the CO_2_/N_2_ separation performance is significantly enhanced under dilute CO_2_ conditions. However, as the CO_2_ feed ratio increases, the separation performance declines and shifts below the upper bound. This trend is attributed to the reduction in CO_2_ solubility associated with saturation of the adsorption sites, together with the diminished diffusivity contrast between CO_2_ and N_2_ at higher CO_2_ concentrations. For CO_2_/CH_4_ separation, although the mixed-gas data points remain below the single-gas values across all compositions due to steric hindrance, they still reside in the high-performance region of the plot. These results underscore the immense potential of A520 (ME) membranes for CO_2_ separation, particularly in dilute CO_2_ streams, where they outperform most existing polymeric and inorganic materials.

To investigate the long-term stability of the membrane under practical industrial operating conditions, the A520 (ME) membrane was further subjected to an extended mixed-gas permeation test. The experiment was conducted at a feed pressure of 3 bar with a CO_2_/N_2_ molar ratio of 50/50 and an operating temperature of 35 °C. The membrane was continuously operated for 120 h, during which gas permeance and separation factor were measured every 24 h. The results are presented in Figure 8. Throughout the testing period, the N_2_ permeability remained nearly constant, and no significant performance deterioration was observed, indicating that the membrane maintained excellent structural integrity and mechanical stability under prolonged pressure-driven operation. In contrast, the CO_2_ permeability exhibited a gradual decline, decreasing from 38.7 Barrer at the beginning of the test to 26.0 Barrer after 120 h of continuous operation. Correspondingly, the CO_2_/N_2_ separation factor decreased from 60.8 to 42.

**Figure 8:**
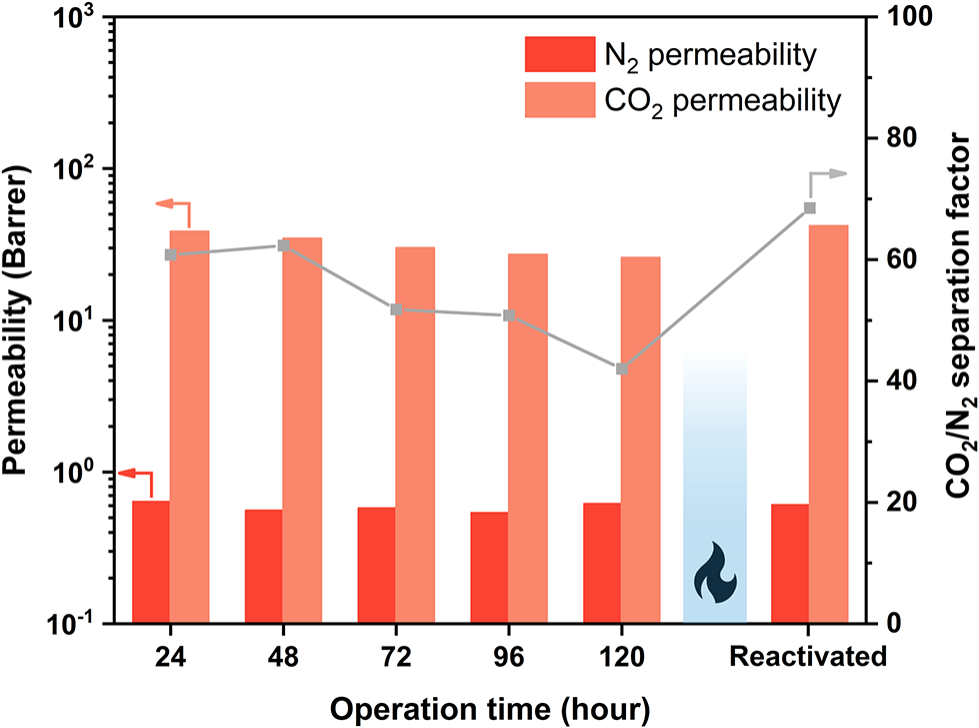
Stability test of the A520 (ME) membrane under an equimolar CO_2_/N_2_ feed mixture at 3 bar and 35 °C. The CO_2_ and N_2_ permeabilities as well as the CO_2_/N_2_ separation factor were measured at 24-h intervals over a 120-h continuous operation period. After 120 h, the membrane was thermally reactivated under vacuum at 150 °C for 1 h, and its performance was re-evaluated under the same operating conditions. Throughout the test, the N_2_ permeability remained nearly constant, while the CO_2_ permeability gradually decreased, resulting in a decline in the separation factor. Following thermal activation, both the CO_2_ permeability and the separation factor recovered, with the separation factor exceeding its initial value, demonstrating the excellent regenerability and long-term stability of the membrane.

This gradual reduction in performance is likely attributable to the adsorption of trace amounts of water vapor present in the feed gas or gas delivery lines within the membrane pores. Nevertheless, upon completion of the 120 h test, the membrane was thermally reactivated under high vacuum at 150 °C for 1 h. Following reactivation, the CO_2_ permeability recovered to 42.0 Barrer, while the CO_2_/N_2_ separation factor increased to 68.5, both exceeding their initial values measured prior to the long-term test. These results demonstrate that the observed performance decline is reversible and primarily associated with the accumulation of adsorbed species rather than irreversible structural degradation of the membrane.

## Conclusions

In summary, we have successfully developed a robust seeded-growth protocol to fabricate continuous polycrystalline A520 (Al-fumarate) MOF membranes on α-alumina substrates. A key finding of this study is the critical role of the post-synthetic activation process in preserving membrane integrity. Methanol exchange (ME) prior to thermal activation effectively suppressed drying-induced lattice distortion caused by capillary stress during water evaporation, resulting in a well-intergrown A520 membrane with a thickness of approximately 3 μm. The resulting A520 (ME) membranes exhibited excellent molecular sieving performance, with ideal CO_2_/N_2_ and CO_2_/CH_4_ selectivities of 71.0 and 112, respectively. Mixed-gas permeation experiments revealed strong composition-dependent separation behavior, with the CO_2_/N_2_ separation factor reaching 153 at a 20% CO_2_ feed concentration, exceeding the 2019 Robeson upper bound. Moreover, the membrane maintained stable separation performance during a 120 h mixed-gas permeation test and recovered its performance after thermal reactivation, demonstrating excellent operational stability and regenerability. Combined GCMC simulations and transport–sorption decoupling analysis provided insights into the mixed-gas transport mechanism. The enhanced CO_2_/N_2_ separation originates from the preferential adsorption of CO_2_ and the strong dependence of N_2_ diffusivity on gas composition. Although increasing N_2_ partial pressure enhances N_2_ uptake, the corresponding reduction in N_2_ diffusional mobility indicates that additional N_2_ molecules occupy less favorable adsorption environments within the confined A520 channels, resulting in restricted molecular transport. In contrast, CO_2_/CH_4_ separation is mainly governed by the intrinsic molecular sieving capability of the A520 framework, where the larger kinetic diameter of CH_4_ leads to stronger diffusion limitation under competitive adsorption conditions. As the first systematic study of pristine A520 MOF membranes, this work highlights the importance of controlled activation strategies and provides fundamental insights into competitive adsorption and confined diffusion for advanced gas separation applications.

## Supplementary Material



## Notes

Photographic images and SEM characterizations of the α-alumina substrates; schematic illustration of the gas permeation setup; SEM images, PXRD patterns, TGA curves, adsorption isotherms, and pore size distribution analyses of A520 powders; comparison of GCMC-simulated single-gas adsorption isotherms with experimental measurements; and benchmark tables comparing CO2/N_2_ and CO_2_/CH_4_ separation performance of various MOF membranes (PDF)
