# Supplementary material for: Activation-Controlled Structural Integrity in A520 MOF Membranes for Efficient CO2/N2 and CO2/CH4 Separation
Source: ACS Appl Mater Interfaces. 2026 Jul 8;18(28):38960–71. doi: 10.1021/acsami.6c09893 (PMC13397478; doi:10.1021/acsami.6c09893)
Supplement: Supplementary file 1 [file am6c09893_si_001.pdf]

Supporting Information

for

Activation-Controlled Structural Integrity in A520 MOF Membranes for Efficient CO_2/N_2 and CO_2/CH_4 Separation

Li-Tang Chi[a], Li-Wei Hsiao[a], Chia-Hui Chuang[a], and Dun-Yen Kang[a,b,c]*

[a]*Department of Chemical Engineering, National Taiwan University, No. 1, Sec. 4, Roosevelt Road, Taipei 106319, Taiwan*

[b]*Center of Atomic Initiative for New Materials, National Taiwan University, No. 1, Sec. 4, Roosevelt Road, Taipei 106319, Taiwan*

[c]*Center of Condensed Matter Sciences, National Taiwan University, No. 1, Sec. 4, Roosevelt Road, Taipei 106319, Taiwan*

Dun-Yen Kang *E-mail: dunyen@ntu.edu.tw

Supporting Figures

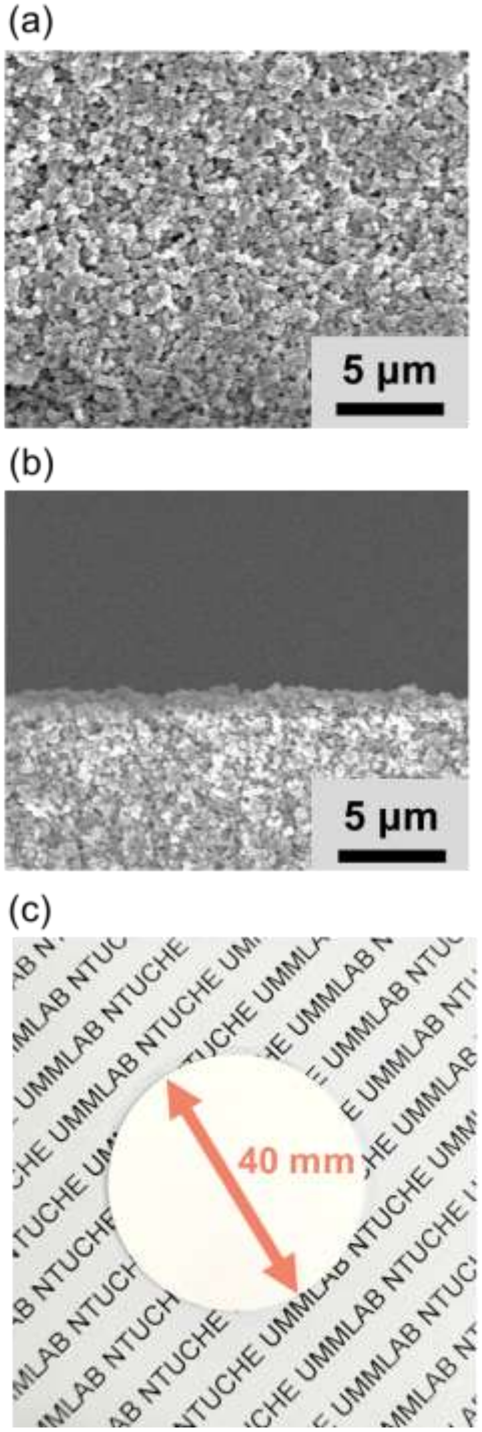

Figure S1. (a) Top-view and (b) cross-sectional SEM images of the porous α-alumina substrate used for membrane growth. (c) Photographic image of the substrate.

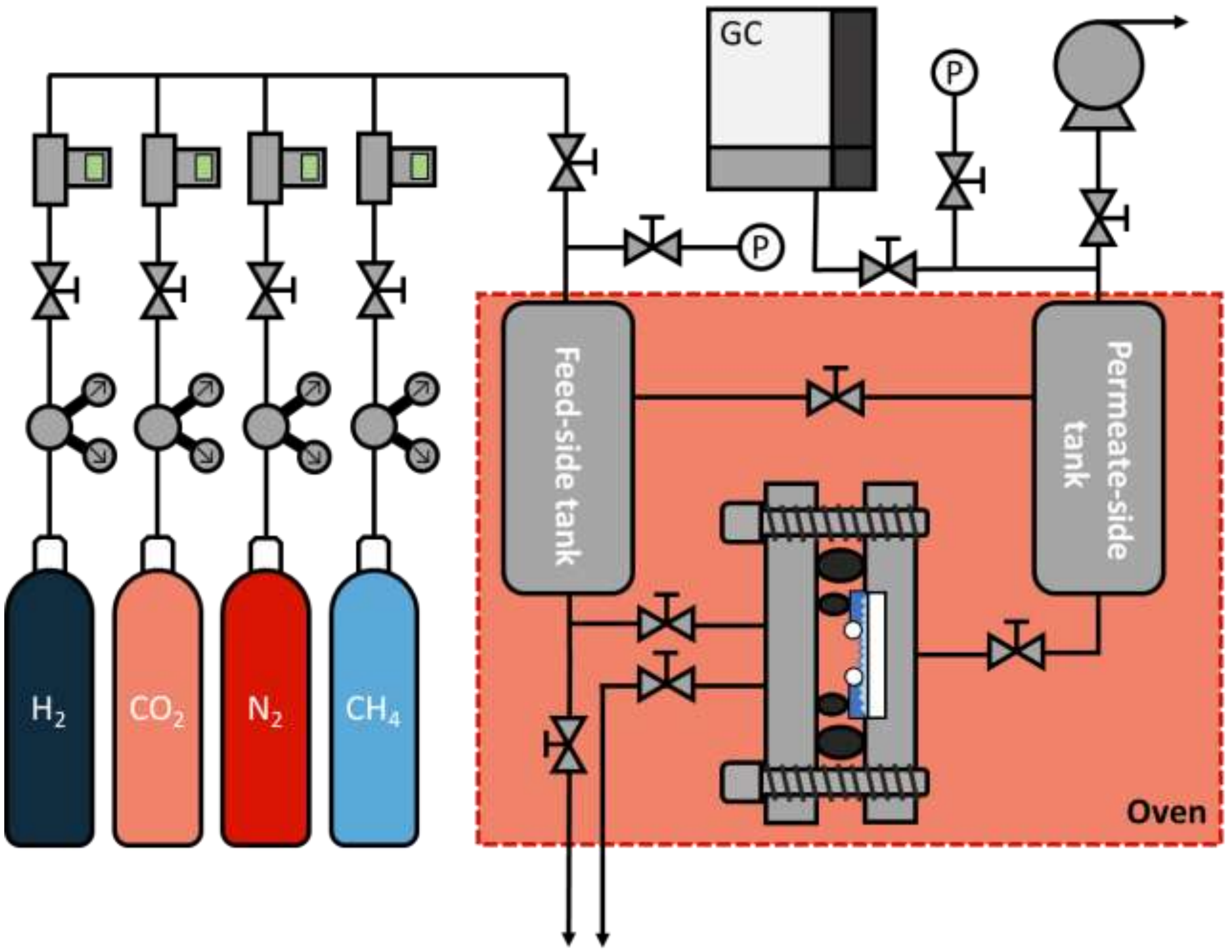

Figure S2. Schematic illustration of the gas permeation system. Gas permeability was determined using the constant-volume method, in which the downstream pressure increase was monitored by a pressure transducer connected to a calibrated fixed-volume chamber. The upstream feed composition was controlled using mass flow controllers (MFCs), while the downstream permeate composition was analyzed by gas chromatography to determine the separation factor (S.F.). In the illustration of the membrane cell assembly, the black components represent O-rings, the cyan regions indicate aluminum tape, the white sections denote epoxy resin used for edge sealing, and the light-blue layer corresponds to the A520 MOF membrane.

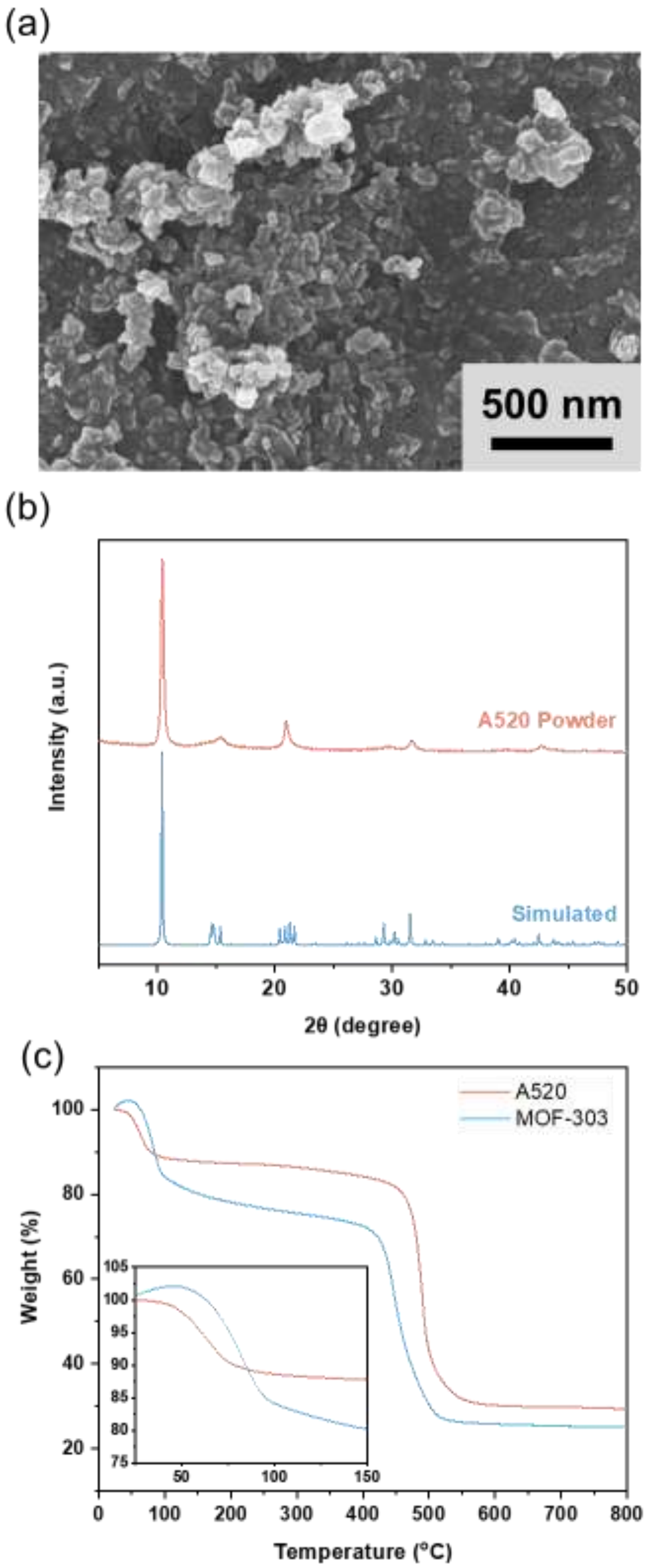

Figure S3. (a) SEM image, (b) powder XRD pattern, and (c) TGA curve of the synthesized A520 powder, compared with MOF-303. A simulated powder diffraction pattern is included in (b) for comparison.

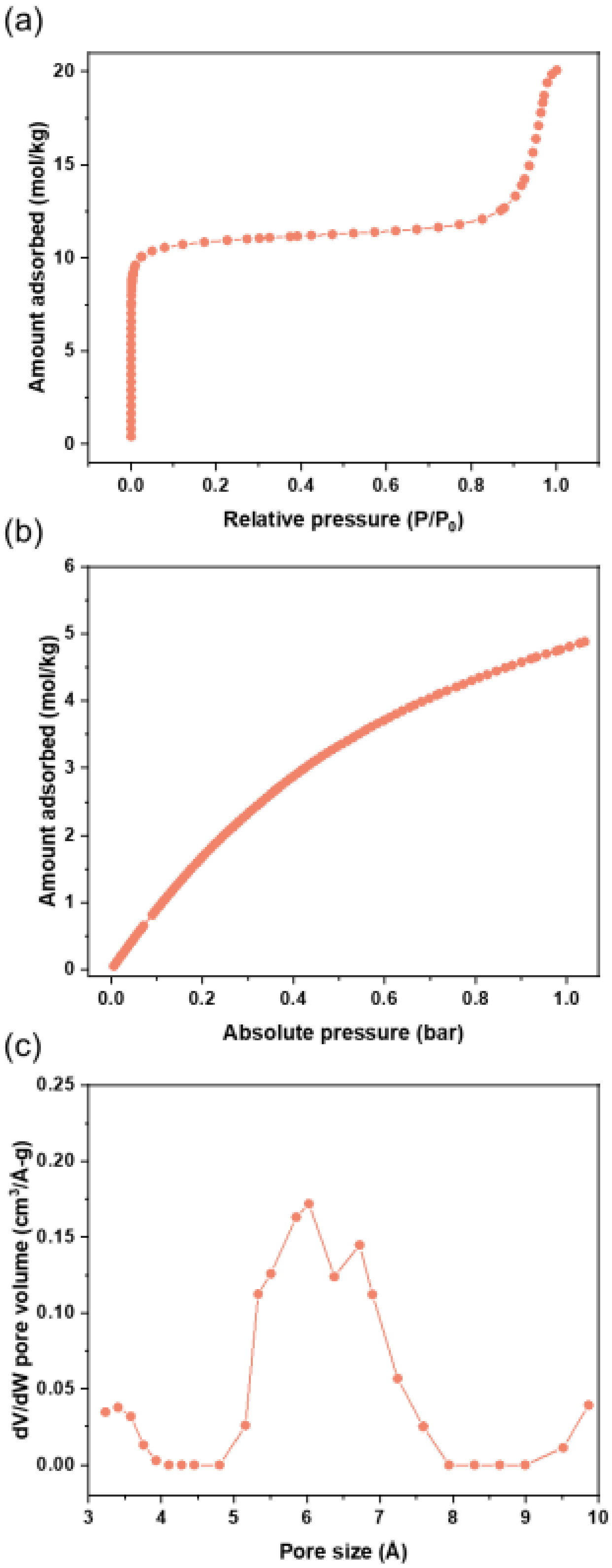

Figure S4. (a) N_2 adsorption isotherms measured at 77 K and (b) CO_2 adsorption isotherms measured at 273 K for A520. (c) Pore size distribution of A520 derived from CO_2 adsorption data using a DFT-based analysis method.

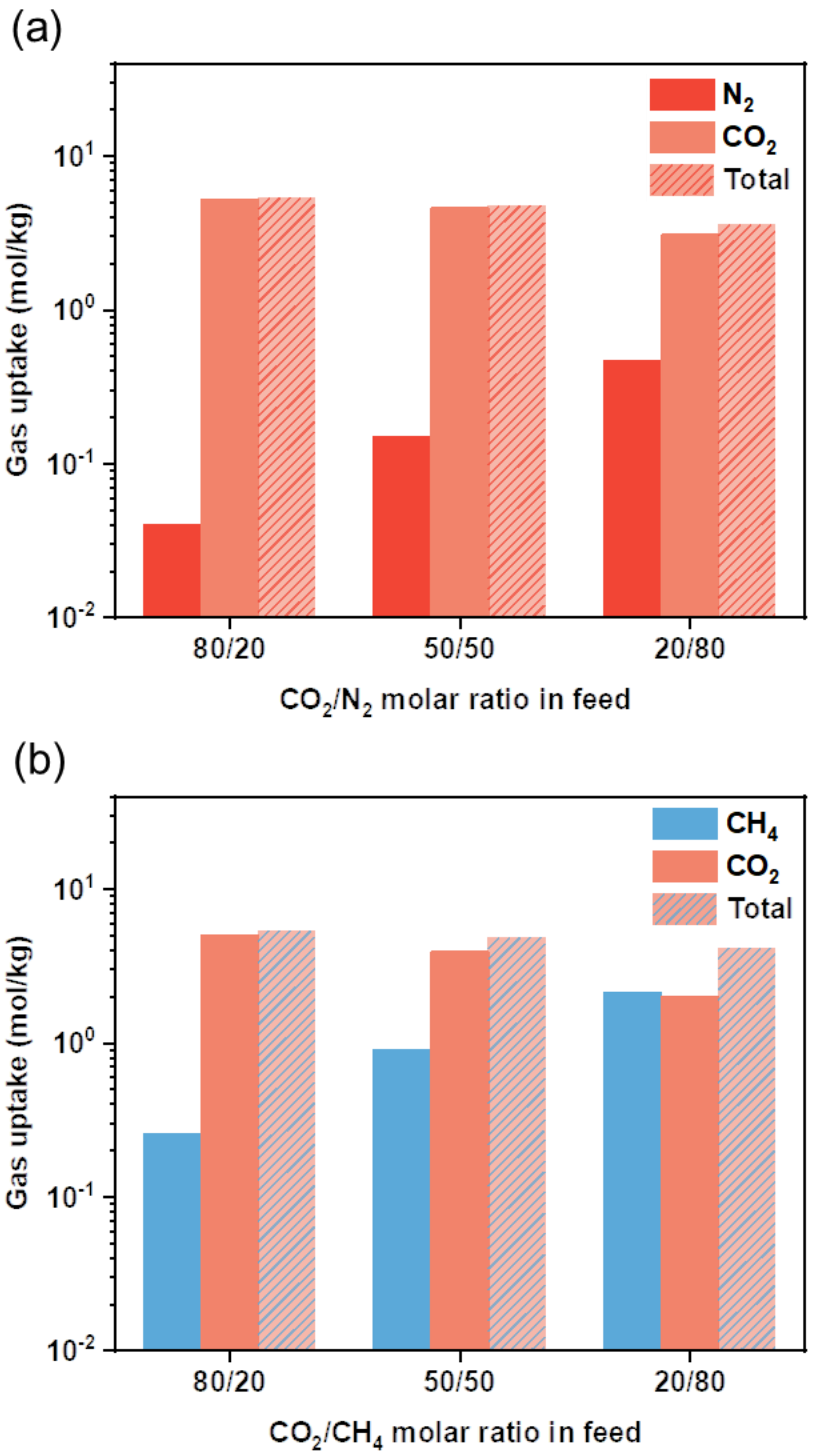

Figure S5. Comparison of mixed-gas adsorption behavior based on GCMC-calculated uptake values. The simulated solubility coefficients were converted into adsorption uptakes, and the total adsorption uptake was included to evaluate changes in gas loading under (a) CO_2/N_2 and (b) CO_2/CH_4 mixed gas conditions.

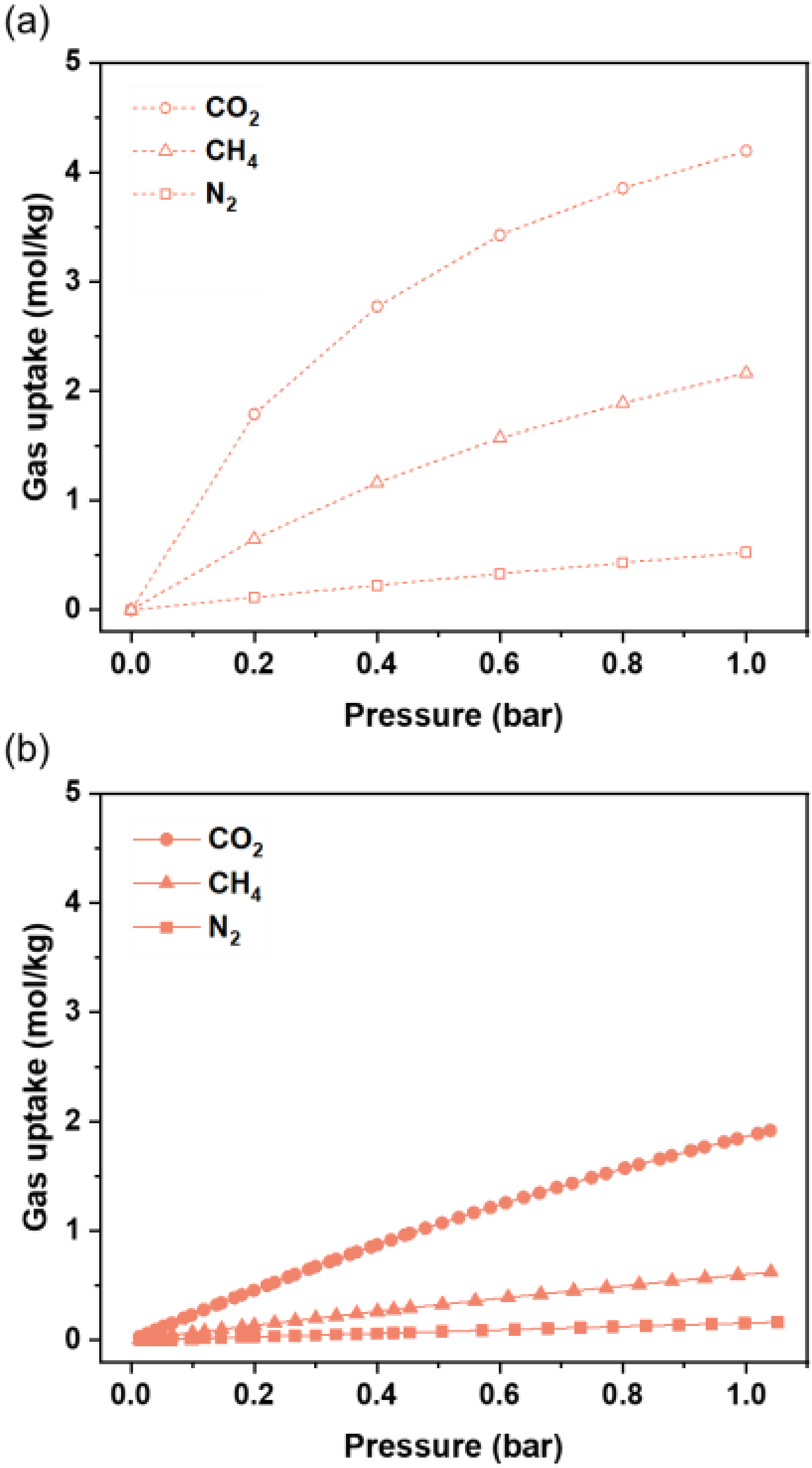

Figure S6. Comparison of (a) GCMC-simulated and (b) experimental single-gas adsorption isotherms of CO_2, N_2, and CH_4 in A520. The adsorption simulations and measurements were conducted at 35 °C and up to 3 bar. Solid lines represent the GCMC simulation results, whereas symbols represent the experimental data.

Table S1. CO_2 permeabilities (P_{CO2}) and CO_2/N_2 separation factors (S.F.) of various MOF membranes.

Materials	X_{CO2}	Thickness (μm)	P_{CO2} (Barrer)	S.F. or Ideal Selectivity* (-)	Reference No. in Main Text
A520 (ME)	Single gas	3	67.2	71.0	**This work**
A520 (ME)	0.2	3	112	153	**This work**
A520 (ME)	0.5	3	62.1	59.1	**This work**
A520 (ME)	0.8	3	44.2	18.6	**This work**
A520 (as made)	Single gas	3	23.8	0.803	**This work**
2nd growth MOF-303	Single gas	3	30.3	62.0	**13**
MOF-303(P6F4)	0.2	5	17.2	170	**13**
MOF-303(P6F4)	0.5	5	17.3	236	**13**
ZIF-69	0.5	40	2050	6.30	**41**
IRMOF-1	0.874	14	8610	410	**42**
UiO-66	0.5	3.5	245	21.4	**43**
MOF-5	0.88	14	18800	70.0	**44**
CAU-1	0.90	3	11600	22.8	**45**
ZIF-62	0.5	70	2600	34	**46**
UTSA-280	0.2	10	17.6	611	**47**
MOF-303(P6F4)	Single gas	5	124	72.1	**13**
MOF-303(P7F3)	Single gas	5	50.6	76.0	**13**
MIL-160	Single gas	15	1350	7.93	**13**
ZIF-69-ideal	Single gas	40	2820	2.20	**41**
IRMOF-1-ideal	Single gas	14	6270	0.820	**42**
Sod-ZMOF-1-ideal	Single gas	37.5	70.5	8.70	**48**
UiO-66-ideal	Single gas	3.5	209	31.3	**43**

CAU-1-NH_2-ideal	Single gas	3	15500	26.2	**45**
MMOF-ideal	Single gas	20	53.7	5.00	**49**
ZIF-8(RHT)-ideal	Single gas	0.55	65.7	37.3	**50**
ZIF-62-ideal	Single gas	72	2050	23.7	**46**
CAU-10-H-ideal	Single gas	4	500	42.0	**51**
ZIF-8-ideal	Single gas	0.5	1710	2.10	**52**
MIL-160-ideal	Single gas	3	1520	33.3	**9**
CAU-10-PDC-H (7:3)-ideal	Single gas	10	1080	25.1	**53**

*S.F. values correspond to mixed-gas permeation measurements, whereas ideal selectivities correspond to single-gas permeation measurements.

Table S2. CO_2 permeabilities (P_{CO2}) and CO_2/CH_4 separation factors (S.F.) of various MOF membranes.

Materials	X_{CO2}	Thickness (μm)	P_{CO2} (Barrer)	S.F. or Ideal Selectivity* (-)	Reference No. in Main Text
A520 (ME)	Single gas	3	67.2	112	**This work**
A520 (ME)	0.2	3	42.5	122	**This work**
A520 (ME)	0.5	3	29.2	55.1	**This work**
A520 (ME)	0.8	3	27.1	99.4	**This work**
A520 (as made)	Single gas	3	23.8	0.613	**This work**
2nd growth MOF-303	Single gas	3	30.3	133	**13**
MOF-303(P6F4)	0.2	5	23.1	431	**13**
MOF-303(P6F4)	0.5	5	21.5	393	**13**
CAU-10-H	0.5	4	2240	50	**51**
ZIF-8-RHT	0.5	0.550	77.2	28.8	**50**
ZIF-62	0.5	70	2638	36.0	**46**
ZIF-7-8	0.5	0.5	22.4	24.5	**54**
$Co_3(HCOO)_6$	0.5	2.25	64700	12.6	**55**
UiO-66	0.5	3.5	436	9.3	**43**
UTSA-280	0.2	10	49.6	42.6	**47**
IRMOF-1	0.815	14	10700	328	**42**
MOF-303(P6F4)	Single gas	5	124	120	**13**
MOF-303(P7F3)	Single gas	5	50.6	172	**13**
MIL-160	Single gas	15	1350	10.8	**13**
CAU-1-NH_2-ideal	Single gas	2.5	9857	14.8	**45**
ZIF-94-ideal	Single gas	4	28.4	37.7	**56**
MIL-160-ideal	Single gas	3	2047	71.0	**9**

CAU-10-H-ideal	Single gas	4	507	95	**51**
ZIF-8-RHT-ideal	Single gas	0.550	59.1	24.7	**50**
ZIF-62-ideal	Single gas	70	2047	27.0	**46**
UiO-66-ideal	Single gas	3.5	210	12.5	**43**
$Co_3(HCOO)_6$-ideal	Single gas	2.25	73900	5.42	**55**
CAU-10-PDC-H (7:3)-ideal	Single gas	10	1080	46.1	**53**

*S.F. values correspond to mixed-gas permeation measurements, whereas ideal selectivities correspond to single-gas permeation measurements.
